# Supplementary material for: End Sequence Analysis Toolkit (ESAT) expands the extractable information from single-cell RNA-seq data
Source: Genome Res. 2016 Oct;26(10):1397–410. doi: 10.1101/gr.207902.116 (PMC5052061; doi:10.1101/gr.207902.116)
Supplement: Supplemental Material [file supp_gr.207902.116_Supp_Materials.tar.gz › ESAT-esat_v0.1/doc/html/functions_vars_b.html]

ESAT: Class Members - Variables


ESAT


- Main Page
- Related Pages
- Packages
- Classes
- Files

- Class List
- Class Index
- Class Hierarchy
- Class Members

- All
- Functions
- Variables
- Enumerator

- a
- b
- c
- d
- e
- f
- g
- h
- i
- k
- l
- m
- n
- o
- p
- q
- r
- s
- t
- u
- v
- w


All Classes Namespaces Files Functions Variables Enumerator Pages

### - b -

- BACKGROUND
  : broad.pda.seq.rap.SkellamKLPeakCaller
- backgroundData
  : broad.pda.seq.protection.TwoSampleScanSkellamPeakCaller
- backgroundName
  : broad.pda.seq.protection.TwoSampleScanSkellamPeakCaller
- backgroundProcessor
  : broad.pda.seq.protection.TwoSampleScanSkellamPeakCaller
- backgroundScanPvalues
  : broad.pda.seq.protection.TwoSampleScanSkellamPeakCaller
- BAM\_EXTENSION
  : broad.pda.seq.rap.CollectAnnotationEnrichments
- basicGenomicAnnotationfactory
  : broad.core.annotation.AnnotationFactoryFactory
- BbvI
  : broad.core.primer3.RestrictionEnzyme
- BceAI
  : broad.core.primer3.RestrictionEnzyme
- bedFactory
  : broad.core.annotation.AnnotationFactoryFactory
- bedGraphFactory
  : broad.core.annotation.AnnotationFactoryFactory
- binomialWindowScores
  : broad.pda.seq.protection.MultiSampleScanPeakCaller
- blocks
  : umms.core.annotation.BasicAnnotation
- BpmI
  : broad.core.primer3.RestrictionEnzyme
- BpuEI
  : broad.core.primer3.RestrictionEnzyme
- BsgI
  : broad.core.primer3.RestrictionEnzyme
- BsmFI
  : broad.core.primer3.RestrictionEnzyme
- BtgZI
  : broad.core.primer3.RestrictionEnzyme
- BUFFER
  : broad.pda.seq.rap.rna.CollectRnaSeqMetrics
- BUFFER\_SIZE
  : broad.core.sequence.AbstractFastaParser
  , broad.core.sequence.FastaQualityBinaryIO


---

Generated on Fri Aug 15 2014 15:11:17 for ESAT by  

 1.8.7
